# Synergistic effect of defocus incorporated multiple segment glasses and repeated low level red light therapy against myopia progression

**DOI:** 10.1038/s41598-024-81363-5

**Published:** 2025-02-01

**Authors:** Yan Yang, Shenghong Liu, Wenwen Gao, Lei Wang, Na Liu, Shiying Zhang, Lianjing Yang, Lingyun Cheng

**Affiliations:** 1 Kangming Eye Hospital, Taiyuan, China; 2 Department of Ophthalmology, Jacob’s Retina Center at Shiley Eye Institute, University of California San Diego, San Diego, La Jolla, CA USA

**Keywords:** Myopia control, Defocus incorporated multiple segment (DIMS) lenses, Repeated low-level red-light (RLRL), Combination therapy of DIMS and RLRL, Changes in axial length, Changes in ocular refraction, Medical research, Outcomes research

## Abstract

Defocus incorporated multiple segment (DIMS) lenses and repeated low-level red-light (RLRL) are used to retard myopia progression. However, it is currently unknown if there is a synergistic effect of the two interventions. In the current study, 190 school-aged children with myopia (380 eyes) were studied for the change in axial length (AL) over nearly one year of follow-up. Of 380 eyes, 170 eyes wore DIMS lenses, 80 eyes had RLRL therapy, and 130 eyes had both interventions (DIMS_RLRL) for myopia control. AL changes were calculated at each follow-up visit by subtracting the baseline measurements and normalized to yearly changes in mm. AL changes as a primary outcome were analyzed in a generalized linear mixed model to compare effect sizes of myopia control among three interventions while adjusting for age, sex, baseline axial length, and follow-up length. Participants had a mean age of 9.84 ± 2.63 years old, mean AL of 24.49 ± 1.20 mm, mean SER of -2.90 ± 2.08 diopters, and mean follow-up time of 301 ± 91 days. By the end of the study, the adjusted mean yearly axial change with combination therapy was − 0.13 mm, -0.04 mm for the eyes with RLRL alone, and 0.16 mm for the eyes with DIMS lenses alone (*p* < 0.0001). Combination therapy of DIMS and RLRL has significantly greater effect size in controlling myopia progression than either RLRL alone (*p* = 0.0009) or DIMS alone (*p* < 0.0001).

## Introduction

With the proliferating of digital media and increasingly prolonged near work^1,2^, the prevalence of myopia in school students has been increasing over the past decades^3^. In East Asia, the myopia rate among primary school students has significantly increased. For instance, the myopia prevalence rate in age 12 students was about 30% during the 1990s and increased to 65% in the 2010s^3^. It is known that the axial length of the myopic eye tends to grow rapidly during the school years^4^; and a higher degree of myopia at an early age is a clear risk factor for future developing myopic retinal degeneration^5^. Degenerative myopia is the number one eye disease leading to irreversible low vision and blindness in the Chinese population^6,7^. Due to growing knowledge about the negative impact of myopia on personal life and social well-being (https://myopiainstitute.org/wp-content/uploads/2020/10/Myopia_report_020517.pdf), management efforts to prevent and reduce the progression of myopia in schoolchildren have been flourishing^8^. Orthokeratology^9^, defocus incorporated multiple segment (DIMS) glasses^10^, and recently repeated low-level red-light (RLRL)^11^ are a few of the options. Low concentration atropine eye drops have also been effective in controlling myopia progression^12^ but less implemented in school students due to its side effects of light sensitivity and near blur.

Though these therapies have been practiced to slow myopia progression, it is largely unknown if these therapies have synergistic effects on myopia control. Since RLRL was reported to be effective in controlling myopia progression^13,14^, its application is broadening due to the good acceptability from patients and the parents. One of its advantages is that RLRL therapy can be completed at home for only a few minutes and does not appear to have significant side effects^11,13^. Many myopic children and their parents have requested this therapy or added this therapy to existing myopia control. The aim of this study is to find out if there exists any synergistic effect from the combination of RLRL and DIMS on myopia control in school-aged children.

## Materials and methods

Study Design: This is a retrospective cohort study. This study adhered to the tenets of the Declaration of Helsinki (as revised in 2013). The study protocol was reviewed and approved by the Institutional Review Board/Ethics Committee of Kangming Eye Hospital, Taiyuan, China. Informed consents were waived as all data used for this project were deidentified. Since April of 2022, outpatient clinic records of school-aged children who visited for myopia control and adopted one of these three interventions (DIMS, RLRL, or the combined DIMS_RLRL) for at least 6 months and up to 500 days (cutoff) were retrieved for the analysis. Primary visit and subsequent follow-up visit totaled 1570 observational records for 190 patients (DIMS patients *n* = 85, RLRL patients *n* = 40, and the combined therapy DIMS_RLRL *n* = 65). The primary outcome measurement is the axial change in millimeters per year, which was calculated from subtracting baseline axial length by that at follow-ups. The difference was then divided by days of follow-up and normalized to a yearly change. Longitudinal repeated measurements on each patient enhance the accuracy of axial assessment over time and allow for better quantification of the effect size exerted by myopia preventative interventions. We did not exclude patients based on a higher degree of myopia because RLRL has been reported to have strong efficacy among patients with high myopia^15^. In the current study, there were 7 patients and 12 eyes with AL > = 26.5 mm (high myopic eyes^16^) in the RLRL group, three patients and 6 high myopic eyes in the DIMS_RLRL group, and no high myopic eyes in the DIMS group. Study Subjects: A cohort of 190 students, aged from 5 to 16 years, comprise the study population. All patients were from the same regional eye hospital and used the same protocols for intervention and follow-ups. Before the beginning of intervention, all patients went through comprehensive ophthalmic examination, including best corrected visual acuity (BCVA), eye movement, intraocular pressure (IOP), slit-lamp microscopy of the anterior segment of the eye globe, and indirect ophthalmoscopy of the fundus. No abnormal findings other than myopic refractive errors were present in this cohort. At initial and subsequent follow-up, IOLMaster (Car Zeiss Meditec AG, Jena, Germany) or NIDEK AL-Scan (NIDEK Inc., Aichi, Japan) was performed to record eye globe axial length. Six measurements were averaged for each visit report. Spherical refractive error was measured by NIDEK ARK-1 auto refractometer (NIDEK Inc., Aichi, Japan) at the initial visit and subsequent follow-ups. Cycloplegia was only for initial measurement or upon the need to change glasses prescription.

Intervention: Students wore DIMS glasses (DIMS group) or single-vision glasses (RLRL group) for their daily routine. For the RLRL group and the DIMS_RLRL group, students took two sessions of low-level red-light stimulation per day without their DIMS glasses or single-vision glasses. Each session lasted for 3 min and a minimum of 4 h between two sessions. Three types of devices were used for the RLRL stimulation: SECONEE (model sky-11201a), YISHILIANG, and STELLEST. All three devices emit low-level red light with a wave length of 650 ± 10 nm from semiconductor laser diodes. The average power used was 1.15 ± 0.31 milliwatts.

Statistical Analyses: The primary outcome measurements, axial changes in millimeters per year, at each visit were used as a response variable in a generalized linear mixed model to model the effect size of myopia interventions while adjusting for age, sex, baseline axial length, and days of follow-up. Each patient had more than one visit and both eyes in the study; eye was nested in patient coding and used as a random effect in the linear mixed model. For subsequent post-hoc comparisons among the 3 intervention groups, Student’s t all pairwise was used. For tabulating, baseline ocular parameters were expressed as mean and standard deviation or fraction/percentage for discrete and binary data. Spherical equivalent refraction (SER) was calculated as the sum of the spherical diopter and half of the cylindrical diopter. All statistical analyses were two-sided and at the 5% significance level. Jumper statistical software was used throughout the analysis (JMP, version 16; SAS Institute Inc., Cary, NC).

## Results

Baseline Characteristics: Hundred ninety students and 380 eyes were studied. The age of the patients was from 5 to 16 years old. Axial length ranged from 21.42 to 28.12 mm with a median of 24.35 mm. Spherical equivalence fraction ranged from − 0.5 to -10.5 diopters with a median of -2.25 diopters, and astigmatism ranged from 0.5 to -3.0 diopters with a mean of -0.63 diopters and a median of -0.5 diopters. Average cornea power (ACP) was calculated from K1 and K2 values. ACP ranged from 38.9 to 47.61 diopters. For 190 patients, there were 785 visits and an average of 4.13 visits for each patient. Baseline information of patients and groups was presented in Table 1.

**Table 1:** Baseline characteristics of the participants.

Intervention	Sample size	Age	Sex	AL	ACP	SER	IOP	BCVAlogMAR	Follow-up Days
RLRL	40	9.85 ± 2.83	F, 52%	24.25 ± 1.78	43.62 ± 1.34	-2.68 ± 2.82	16 ± 2.65	0.015 ± 0.036*	324 ± 84*
DIMS	85	10.37 ± 2.45	F, 49%	24.35 ± 0.89	43.53 ± 1.45	-2.69 ± 1.53	16.15 ± 2.85	0.004 ± 0.019	301 ± 94
DIMS_RLRL	65	9.00 ± 2.39*	F, 34%**	24.60 ± 1.08*	43.33 ± 1.55*	-2.93 ± 2.06*	16.07 ± 2.87*	0.004 ± 0.018	289 ± 89
p value		0.016; <0.0001	< 0.05	> 0.05	> 0.05	> 0.05	> 0.05	0.001; 0.002	0.23; 0.045

RLRL: Repeated Low-Level Red-Light; DIMS: Defocus Incorporated Multiple Segment; DIMS_RLRL: Combination of DIMS and RLRL; ACP: Average Cornea Power, Diopter; AL: Axial Length; SER: Spherical Equivalant Refraction, Diopter; IOP: Intra Ocular Pressure, mmHg; BCVAlogMAR: Log of Minimum Angle of resolution for Best Corrected Visual Acuity, each letter has a score value of 0.02 log units.

*: Tukey-Kramer HSD, ordered difference.

**: Contingency Analysis of means for proportions.

**Fig. 1:**
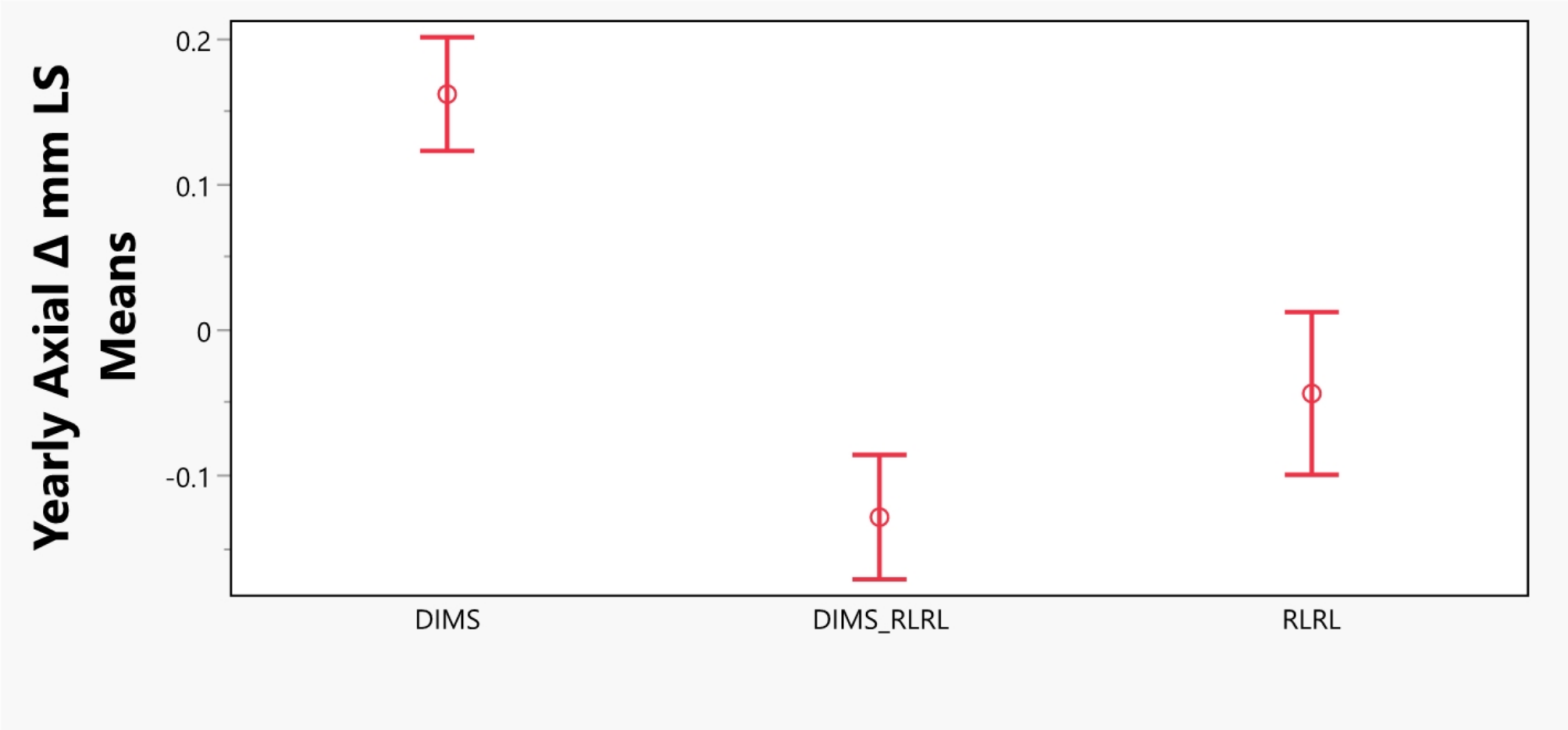
Least Squares Mean Plot. Least squares (LS) mean of yearly axial changes and the intervention groups. The circles represent mean value and the vertical bars represent 95% confidence limits.

Changes in Axial Length: Axial changes in millimeters per year as response and explanatory variables of age, sex, baseline axial length, and days of follow-up were simulated in a generalized linear mixed model. The analysis revealed that there was a significant difference from interventions (*p* < 0.0001) while adjusting for imbalance in age, sex, baseline axial length, and days of follow-up. The least squares mean of axial changes for three interventions is displayed in Fig. 1. Yearly axial change was 0.16 mm for DIMS group, -0.04 mm for RLRL group, and − 0.13 mm for DIMS_RLRL combination group. All pairwise Student’s t tests showed significant differences among them (DIMS vs. DIMS_RLRL, *p* < 0.0001; DIMS vs. RLRL, *p* < 0.0001; DIMS_RLRL vs. RLRL, *p* = 0.0188). Generalized linear mixed model also demonstrated that lower age (β = -0.04, *p* < 0.0001), being female (β = 0.08, *p* = 0.0033), longer baseline AL (β = 0.04, *p* = 0.0019), and longer follow-up days (β = 0.0007, *p* < 0.0001) are associated with greater axial elongation.

**Fig. 2:**
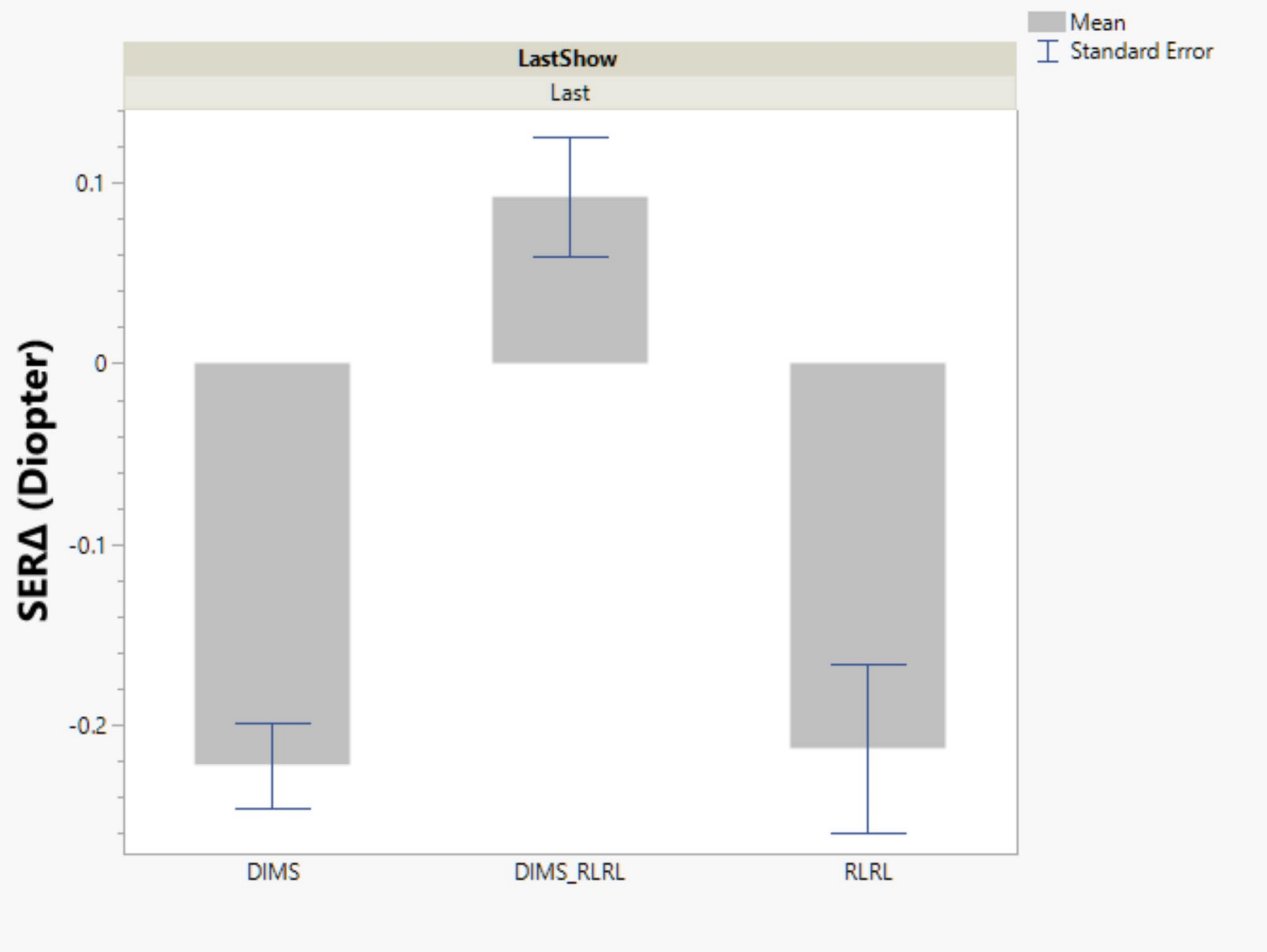
Changes of Spherical Equivalent Refraction (SER) at the last follow-up, stratified by the three interventions.

Changes in Refractive Error: Spherical Equivalent Refraction (SER) was used to calculate SER change between the initial visit and the last visit. Patients in the DIMS_RLRL group had SER reduction of 0.09 ± 0.37 diopters, which is significantly different from SER advancement of -0.21 ± 0.40 diopters for RLRL patients (*p* < 0.0001) and − 0.22 ± 0.31 diopters for DIMS patients (*p* < 0.0001). SER changes were not significantly different between DIMS and RLRL patients (Fig. 2).

Adverse Events: During the intervention and follow-up, no adverse event was observed or reported from patients. IOPs of the patients at the first visit versus that at the last visit were not significantly different for neither of the three-intervention groups (Table 2). Similarly, visual acuity did not change throughout the study period, even though patients in the RLRL group showed a small, statistically significant, but clinically negligible, improvement (-0.012, *p* = 0.039; Table 2).

**Table 2:** Intraocular pressure and visual acuity during the intervention to retard myopia progression.

		RLRL	DIMS	DIMS_RLRL		RLRL	DIMS	DIMS_RLRL
First Visit	IOP	16.00 ± 2.65	16.46 ± 2.47	16.07 ± 2.87	BCVAlogMAR	0.016 ± 0.036	0.004 ± 0.019	0.004 ± 0.018
Last Visit	IOP	16.27 ± 2.61	16.23 ± 2.66	15.99 ± 2.62	BCVAlogMAR	0.004 ± 0.033	0.001 ± 0.024	0.005 ± 0.021
p value		0.54	0.44	0.82		0.039	0.14	0.82

RLRL: Repeated Low-Level Red-Light; DIMS: Defocus Incorporated Multiple Segment; DIMS_RLRL: Combination of DIMS and RLRL; ACP: Average Cornea Power, Diopter; IOP: Intra Ocular Pressure, mmHg; BCVAlogMAR: Log of Minimum Angle of resolution for Best Corrected Visual Acuity; IOP and BCVAlogMAR were presented as mean plus/minus standard deviation.

## Discussion

Myopic eyes have a significantly different ocular growth curve than emmetropic eyes. Myopic eyes experience a fast increase in axial length before age 16 years^17^. In contrast to mild changes of corneal refractive power or lens power, an increase of vitreous chamber depth is prominent^17^. Therefore, the current study used changes in axial length as the key outcome measurement. The multivariate analysis revealed that there might be a synergistic effect from the combination of DIMS and RLRL against myopia progression. Through the study course, children wearing DIMS lenses had a rate of yearly axial change of 0.16 mm, and the yearly rate for children receiving twice daily RLRL was − 0.04 mm. Contrasting to those rates, the yearly rate for children who had combination therapy was − 0.13 mm. Similarly, patients in the DIMS_RLRL group had a SER reduction of 0.09 diopter, while patients in the other two groups had SER advancement (-0.21 diopter for RLRL and − 0.22 diopter for DIMS) during a similar length of follow-up.

The current study did not have a group of single-vision glasses as control due to the nature of retrospective research. However, many clinical trials have demonstrated that either DIMS^10,18–20^ or RLRL^11,15^ alone is effective in controlling myopia progression. Therefore, the use of a single-vision lenses control group is not necessary in the current study setting. The rate of axial change for the DIMS group was 0.16 mm per year (translate to 0.13 mm if 300 days of follow-up), which is similar to the other reports during the similar length of follow-up for eyes wearing DIMS lenses^19,20^ (0.11 mm by Lam CSY; 0.12 mm by Nucci P). The change in axial length for the RLRL group in the current study was − 0.04 mm per year. Such an effect size was on a par with recent two clinical trials, one reported 0.06 mm axial change over 12 month^15^ and the other reported 0.08 mm axial change after one-year RLRL intervention^21^. In the current study, we found a greater myopia-controlling effect size for RLRL than that for DIMS. There is very little information in literature to compare those two interventions. Further prospective studies are needed to shed light on this aspect.

Since both DIMS and RLRL are effective to a certain degree in controlling myopia progression, it is natural to ask if there is a synergistic effect to retard the progression of myopia. There is little information available in the literature except for registration of the clinical trial to be conducted. To the best of our knowledge, the current study is the first to report a synergistic effect from combination of DIMS and RLRL. Patients with the combination therapy had a negative axial change or reduction of axial length during the study course. The finding is encouraging for management of myopia control. Such a combination therapy may be better accepted by students and parents than the proposed combination therapy of DIMS and a low concentration of atropine^20,22^.

Both genetic and environmental factors contribute to myopia development and progression; however, recent years of rapid increase in the prevalence of myopia suggest the connection between increased time of near work and myopia^1^. Eye growth is regulated by visual feedback, and peripheral hyperopic defocus can promote excessive growth of the eye globe, which is evidenced by both animal model research^23^ and a 2-year randomized or retrospective clinical interventional research^24,25^. In a recent publication, both positive and negative peripheral added lenslets slowed down myopia progression in a one-year randomized clinical trial^26^. It seems that our current understanding of myopia progression needs much more investigation before we see a clear path. In contrast to clearer mechanism of the DIMS effect, the mechanism of RLRL intervention is not as clear. The key of the red-light effect is believed to be the spectrum or wavelengths of the light, in which shorter wavelength visible light (blue) in sharper focus triggers axial elongation while longer wavelength light (red) in sharper focus inhibits axial elongation^27^. In addition, some researchers postulate that visible red light can modify cytochrome C oxidase to inhibit scleral remodeling^28^. It seems that DIMS and RLRL exert on different aspects of the pathway for myopia progression; therefore, a synergistic effect is possible.

In summary, the current study demonstrated a synergistic effect from combination therapy of DIMS and RLRL on myopia progression. We acknowledge that this was a retrospective study in which group sampling, follow-up time, and baseline AL among groups were not well balanced. Therefore, we used a multiple linear mixed regression model to adjust for those unbalances. In addition, cycloplegia was not consistently performed at every visit; the presented SER results could be confounded. However, considering regular AL increases by 0.2 mm per year for 6- to 15-year-old myopia schoolchildren^17^, the observed AL changes in the current study attest to the effectiveness of those interventions. It is also encouraging that no adverse event was reported or observed for neither of the three interventions (DIMS, RLRL, and DIMS_RLRL). This is the first such study, and more studies including randomized clinical trials are needed to validate this finding.

## Data Availability

The datasets used and/or analyzed during the current study are available from the corresponding author on reasonable request.
